# Spontaneous Lens Rupture in Congenital Cataract, Presented as Lens Fragment Within the Anterior Chamber

**DOI:** 10.7759/cureus.11699

**Published:** 2020-11-25

**Authors:** Konstantina Sorkou, Maria Eleni Manthou, Soultana Meditskou, Ariadni Gavriilidou, Christos Sioulis

**Affiliations:** 1 Laboratory of Histology and Embryology, Aristotle University of Thessaloniki, Thessaloniki, GRC; 2 2nd Department of Ophthalmology, Aristotle University of Thessaloniki, Thessaloniki, GRC; 3 Department of Ophthalmology, 424 General Military Hospital of Thessaloniki, Thessaloniki, GRC

**Keywords:** congenital cataract, anterior polar cataract, lens rupture, lens fragment

## Abstract

We report the case of a 60-year-old woman with a congenital anterior polar cataract who presented with a lens fragment within the anterior chamber of her left eye, without a medical history of surgery or trauma. Uneventful phacoemulsification with simultaneous removal of the lens fragment followed. Postoperatively, the patient’s visual acuity improved from a perception of light to 20/100. Histological examination of the lens fragment revealed persistent fetal vasculature, as well as fibrous metaplasia and extensive collagenesis. To our knowledge, this is the first report of spontaneous rupture of both the anterior capsule and the lens.

## Introduction

Retained lens fragments constitute a known but relatively uncommon complication of cataract surgery [1-3]. A case of a cone-shaped lens fragment has been described in the anterior chamber of a patient with congenital anterior polar pyramidal cataract, as a remnant after surgery as well [2]. Spontaneous rupture of the anterior lens capsule, but with preserved lens integrity, has also been previously reported in several cases, mostly related to Alport’s syndrome [4-5].

To our knowledge, there are no reports of spontaneous rupture of both capsule and lens, followed by lens fragment detachment in the anterior chamber. We report the case of an adult patient with a congenital anterior polar cataract who presented with anterior capsular rupture, a broken lens, and a translocated lens fragment within the anterior chamber.

## Case presentation

A 60-year-old Caucasian woman visited our outpatient clinic, complaining of the presence of a “foreign body inside her left eye" and an intermittent, sharp pain lasting for three months. Her ocular medical history included no trauma or surgery but a unilateral congenital anterior polar cataract in her left eye. Hypertension, hypothyroidism, headaches under medication, and orthopedic problems were included in her general medical history, without the presence of Alport's syndrome.

**Figure 1:**
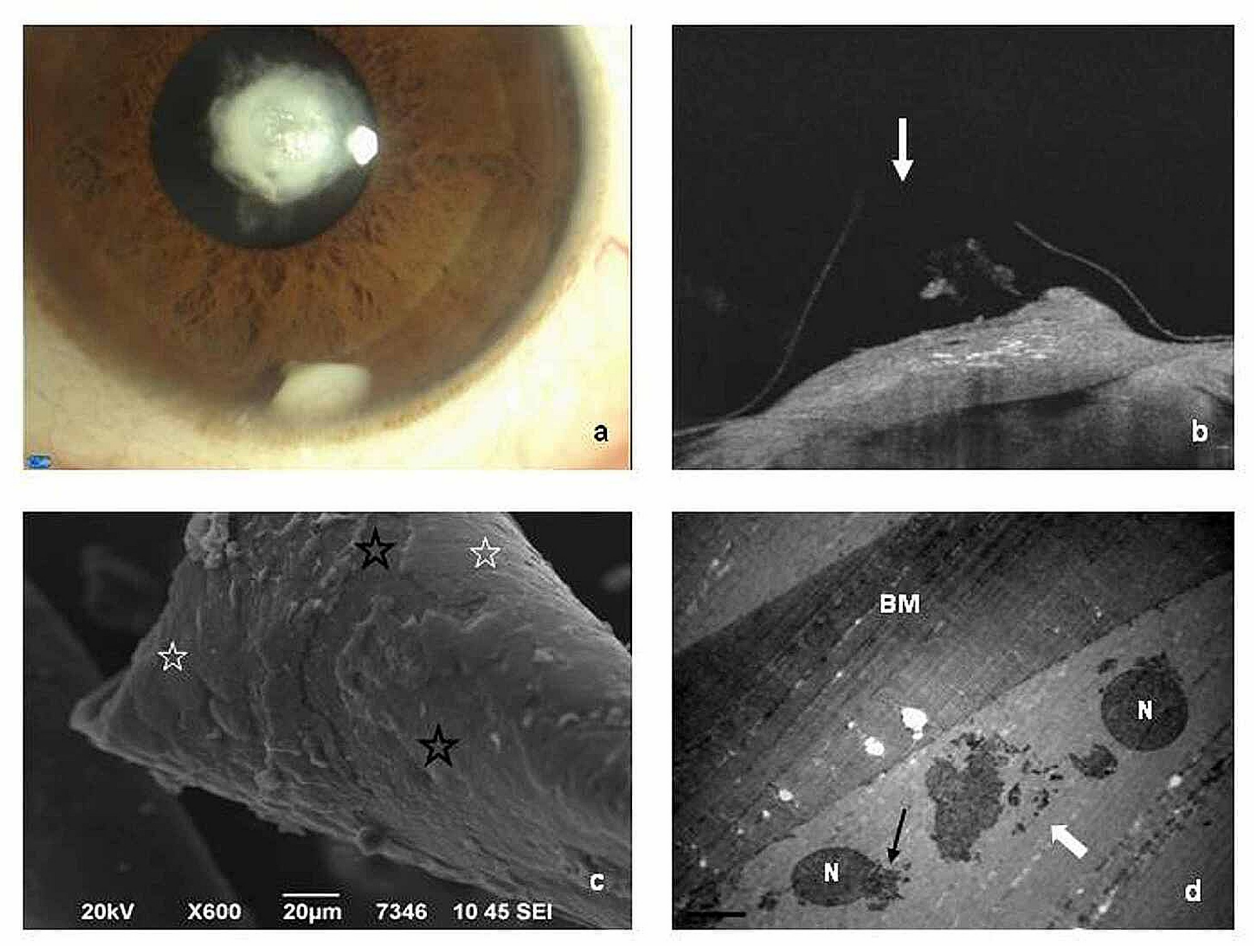
Slit-lamp, OCT, SEM, and TEM photographs a. Slit-lamp photograph (x16) of the anterior polar cataract and the cone-shaped lens fragment. b. OCT image of the anterior capsular rupture (white arrow) and the underlying lens deficiency, corresponding to the cone-shaped translocated lens fragment. c. SEM photograph of aLC (x600). LECs appear flattened (white asterisks). Areas with epithelial cells partly or completely absent, where the underlying basement membrane is visible (black asterisks). d. TEM photograph of aLC (x3000). Destruction of LECs with present only their remnants upon BM. OCT: optical coherence tomography; SEM: scanning electron microscope; TEM: transmission electron microscopy; LEC: lens epithelial cell; BM: basement membrane; N: nucleus

Visual acuity (VA) in the left eye was light perception, improving to counting fingers at 1 meter, with a dilated pupil, whereas in the right eye, the best-corrected VA was 20/20. The intraocular pressure (IOP) was 14 mmHg bilaterally. In slit-lamp examination, an opaque, white, cone-shaped lens fragment was detected in the inferior anterior chamber of the left eye, the basis of which was supplementary related to the central rough surface of the anterior polar cataractic lens. It changed position with eye movements and measured 1.90 x 2.10 mm (Figure 1, panel 1a). Neither cells nor flare in the anterior chamber and no corneal edema was detected. Slit-lamp examination of the other eye revealed no pathological findings.

Optical coherence tomography (OCT) revealed a central rupture of the anterior capsule and a deficiency of the anterior polar cataractic lens, which corresponded to the translocated lens fragment. The cataract remnants on the lens side seemed to play the role of a sealant, not allowing the exudation of lens material (Figure 1, panel 1b). Due to the absence of intraocular inflammation and normal IOP, the patient was scheduled for surgery five weeks later. In the meantime, she had regular weekly re-evaluations, with stable clinical appearance.

The uneventful surgery, performed by an experienced cataract and vitreoretinal surgeon (C.S.), included the removal of the lens fragment and phacoemulsification with intraocular lens insertion within the capsular bag. The most challenging stages were the removal of the small, hard lens fragment through the main incision and the capsulorrhexis, incorporating the centrally ruptured anterior lens capsule, without any complications.

The anterior capsule, which was removed during continuous curvilinear capsulorrhexis, was fixed into 3% glutaraldehyde solution and was then prepared for scanning electron microscope (SEM) and transmission electron microscopy (TEM). The lens fragment was fixed in 10% formaldehyde solution and sections were stained with hematoxylin-eosin, Periodic Acid-Schiff (PAS), Giemsa, and trichrome Masson. The patient signed informed consent. The Ethics Committee of 424 General Military Hospital approved the study (Α.Π. 5122/7-3-2019), according to the tenets of the Declaration of Helsinki.

**Figure 2:**
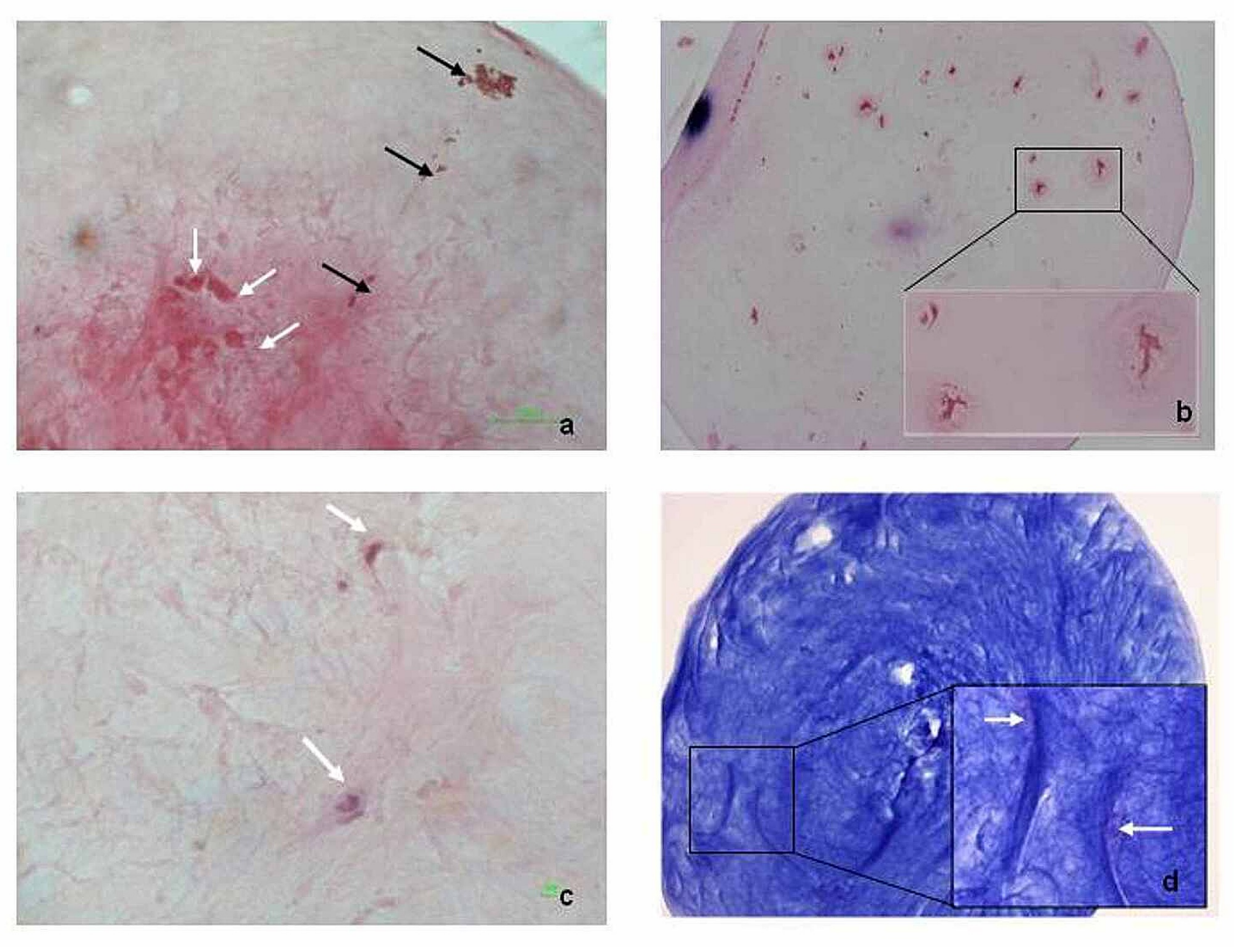
Light microscopy images of the lens fragment a. PAS (x100). Protein accumulation with the formation of Morgagnian globules (white arrows). Linear presence of red blood cells, indicating the persistence of fetal vasculature (black arrows). b. Giemsa (x100, inset x400). Crystalloids formation in the same fragment. c. Giemsa (x400). Spindle-shaped migrating LECs (arrows) with protrusions and myofibroblastic characteristics. d. Masson trichrome (x100, inset x200). Extensive collagen staining (blue) due to fibrous metaplasia of migrating LECs (pink), indicated with arrows. LEC: lens epithelial cell; PAS: Periodic Acid-Schiff

The lens epithelial cells (LECs) of the anterior capsule in many areas of the sample appeared flattened or were completely absent in SEM and TEM (Figure 1, panels 1c-1d). The histological examination of the lens fragment revealed indications of persistent fetal vasculature (PFV) and the presence of degenerative structures such as Morgagnian globules and crystalloids (Figure 2, panels 2a-2b). Fibrous metaplasia of migrating LECs, with extensive collagen formation, was also documented (Figure 2, panels 2c-2d).

During the first postoperative day, the patient demonstrated moderate inflammation in AC (grade 2+) and striae in the cornea, which were gradually improved during the first week. One month later, neither cells nor flare was detected in AC, the cornea was transparent, and the VA reached 20/100. The IOP was stable at 14 mmHg throughout the follow-up period. The patient was satisfied with the absence of subjective symptomatology and her vision improvement.

## Discussion

In electron microscopy observation, the LECs’ flattening seems to be related to cataract degenerative manifestations, which constitute a quite common finding [6]. However, LECs’ destruction is not usually described ultrastructurally and, in this case, is probably attributed to the mechanical friction caused by lens rupture. In an optical microscope, all the described findings are known to be related to cataract pathogenesis [7]; nonetheless, there is no documentation as to how extended these alterations are expected to be. Fibrous metaplasia and collagenesis contribute to the hardening of the smooth lens, but they are not normally expected to result in spontaneous lens rupture and in the detachment of a lens fragment. In the present case, it seems that the rupture was probably caused by the particularly extended alterations.

The histological manifestation of PFV in the patient confirms the pathogenesis of her congenital cataract [7], which is probably correlated with severe degenerative histological findings. Nowadays, surgical intervention before six weeks of age is suggested for a unilateral congenital cataract to prevent amblyopia, strabismus, nystagmus, or glaucoma [8-9]; nevertheless, it was never recommended to our patient.

## Conclusions

Overall, the congenital cataract complication that we came across, as well as the subjective and objective postoperative improvement, is strongly suggestive of the benefits of prompt surgical treatment as soon as the condition is diagnosed even after the recommended critical age when amblyopia is theoretically established.
